# Lack of Transmission of Foot-and-Mouth Disease Virus From Persistently Infected Cattle to Naïve Cattle Under Field Conditions in Vietnam

**DOI:** 10.3389/fvets.2018.00174

**Published:** 2018-07-27

**Authors:** Miranda R. Bertram, Le T. Vu, Steven J. Pauszek, Barbara P. Brito, Ethan J. Hartwig, George R. Smoliga, Bui H. Hoang, Nguyen T. Phuong, Carolina Stenfeldt, Ian H. Fish, Vo V. Hung, Amy Delgado, Kimberley VanderWaal, Luis L. Rodriguez, Ngo T. Long, Do H. Dung, Jonathan Arzt

**Affiliations:** ^1^Foreign Animal Disease Research Unit, Plum Island Animal Disease Center, Agricultural Research Service, USDA, Orient Point, NY, United States; ^2^Plum Island Animal Disease Center Research Participation Program, Oak Ridge Institute for Science and Education, Oak Ridge, TN, United States; ^3^Regional Animal Health Office No. 6, Department of Animal Health, Ministry of Agriculture and Rural Development, Ho Chi Minh City, Vietnam; ^4^STEMMA Laboratory, Veterinary Population Medicine, University of Minnesota, St. Paul, MN, United States; ^5^Monitoring and Modeling, Center for Epidemiology and Animal Health, APHIS, USDA, Fort Collins, CO, United States; ^6^Department of Animal Health, Ministry of Agriculture and Rural Development, Hanoi, Vietnam

**Keywords:** foot-and-mouth disease virus, FMD, carriers, sentinels, transmission, duration of carrier state, viral evolution, phylogenetics

## Abstract

Foot-and-mouth disease (FMD), caused by FMD virus (FMDV; *Aphthovirus, Picornaviridae*), is a highly contagious and economically important disease of cloven-hoofed domestic livestock and wildlife species worldwide. Subsequent to the clinical phase of FMD, a large proportion of FMDV-infected ruminants become persistently infected carriers, defined by detection of FMDV in oropharyngeal fluid (OPF) samples 28 days or more post-infection. The goal of this prospective study was to characterize the FMD carrier state in cattle subsequent to natural infection under typical husbandry practices in Vietnam. Ten persistently infected cattle on eight farms in the Long An province in southern Vietnam were monitored by monthly screening of serum and oropharyngeal fluid samples for 12 months. To assess transmission from FMDV carriers, 16 naïve cattle were intentionally brought into direct contact with the persistently infected animals for 6 months, and were monitored by clinical and laboratory methods. The restricted mean duration of the FMD carrier state was 27.7 months, and the rate of decrease of the proportion of carrier animals was 0.03 per month. There was no evidence of transmission to naïve animals throughout the study period. Additionally, there was no detection of FMDV infection or seroconversion in three calves born to carrier animals during the study. The force of infection for carrier-to-contact transmission was 0 per month, with upper 95% confidence limit of 0.064 per month. Phylogenetic analysis of viral protein 1 (VP1) coding sequences obtained from carriers indicated that all viruses recovered in this study belonged to the O/ME-SA/PanAsia lineage, and grouped phylogenetically with temporally and geographically related viruses. Analysis of within-host evolution of FMDV, based upon full-length open reading frame sequences recovered from consecutive samples from one animal, indicated that most of the non-synonymous changes occurred in L_pro_, VP2, and VP3 protein coding regions. This study suggests that the duration of FMDV persistent infection in cattle may be longer than previously recognized, but the risk of transmission is low. Additional novel insights are provided into within-host viral evolution under natural conditions in an endemic setting.

## Introduction

Foot-and-mouth disease (FMD), caused by FMD virus (FMDV; *Aphthovirus, Picornaviridae*), is a highly contagious and economically important disease of cloven-hoofed domestic livestock and wildlife species worldwide. Acute infection is characterized by loss of appetite, fever, and formation of characteristic vesicles on the feet, udders, and in the oral cavity (1–3). Mortality is generally low, however high morbidity results in economic losses due to decreased production in endemic regions as well as imposed trade restrictions subsequent to outbreaks (4–7).

A large proportion of FMDV-infected ruminants become persistently infected carriers, which is defined by detection of FMDV in oropharyngeal fluid (OPF) samples 28 days or more post infection (8, 9). Prevalence of carriers has been reported to be >50% in cattle (10–12), and 50–70% in African (Cape) buffalo (*Syncerus caffer*) (13–15). African buffalo can be persistently infected for up to 5 years (14), and cattle up to 2 years (16, 17), although many carrier cattle have been reported to clear the infection within 6 months (8, 16, 18–20). Vaccination using a homologous strain is efficient in preventing clinical disease, but does not protect against subclinical or persistent infection (10–12); (18, 20–22). Carriers and non-carriers have similar levels of virus shedding during the preceding acute stage of disease (12, 22) and similar antibody responses in serum (23, 24). In African buffalo, more virulent strains may be more likely to establish persistent infections (15), and a small number of studies have found genetic changes in viruses sampled from carrier cattle compared to outbreak viruses. However, these findings have not been consistent across studies (22, 25, 26). Factors that lead to persistent infection have not been fully elucidated, and it is likely that a combination of host and virus factors contribute to establishment and maintenance of the FMD carrier state.

The role of carrier animals in the epidemiology of FMD in endemic areas remains unclear. The African buffalo is the only species demonstrated to transmit FMDV to susceptible animals during the carrier state, with transmission from carrier buffalo to naïve buffalo taking place within 2 weeks of exposure (27–29). Several studies have demonstrated clinical signs of FMD in cattle exposed to persistently infected African buffalo under experimental conditions, however transmission occurred only after 5–10 months of continuous exposure (28, 30). There are anecdotal reports of carrier cattle being the source of outbreaks in Denmark in 1883–1894 and in the UK in 1922–1924 [reviewed in (31)], as well as in Zimbabwe in 1989 and 1991 (32). Additionally, phylogenetic analyses of carrier and outbreak viruses from Vietnam in 2012–2014 suggested that a carrier strain may have been the source of an FMD outbreak (25, 33, 34). However, FMDV transmission from persistently infected cattle to naïve cattle has not been convincingly demonstrated experimentally, despite numerous attempts (10, 22, 35, 36). A meta-analysis of experimental studies investigating transmission from persistently infected animals within and between species (cattle, buffalo, pigs, or sheep) found an overall transmission rate of 0.0256 infections per carrier per month, with most instances of transmission involving African buffalo carriers (37).

FMD is endemic in South-East Asia, and Vietnam plays an important role in FMD epidemiology in the region due to considerable domestic movements of livestock and through trade with China, Cambodia, Laos, and Thailand (38). Cattle and Asian (water) buffalo (*Bubalus bubalis*) are kept for meat and milk production, and are used for draft to a limited extent. Smallholder (subsistence) farms are common, although some larger commercial operations exist (38). Within the smallholder system, animals are often kept in simple pens or restrained near the home, although grazing in communal pastures is also common (39, 40). A recent targeted surveillance study (33) reported that 22.3% of sampled cattle and Asian buffalo in Vietnam had been infected with FMDV, and 2.4% of sampled animals were persistently infected. Thus, carrier animals are likely present throughout the country, and the extensive movement of animals within Vietnam and between Vietnam and neighboring countries suggests that FMDV carriers may pose a risk as sources of new outbreaks (34). The duration of persistent infection and the risk of transmission from carrier animals have practical implications for animal movement, trade, and FMD control. The primary purpose of this study was to investigate the potential for transmission of FMDV from persistently infected cattle to naïve cattle under typical husbandry conditions in Vietnam. Ancillary goals included quantification of extinction of the carrier state and characterization of within-host viral evolution.

## Methods

### Ethics approval

The work described herein was performed by federal staff of the Department of Animal Health, Ministry of Agriculture and Rural Development, Government of Vietnam. The work occurred and the animals were maintained within facilities that were owned, maintained, or overseen by this division of the federal government; thus, no permits or approvals were required. Additionally, approval was not required for the questionnaire given to farmers per local legislation. All cases described herein occurred spontaneously in domestic cattle with no experimentation, inoculation, or treatment of live animals. No animals were euthanized for the purpose of this study.

### Transmission study design and sample collection

**Figure 1:**
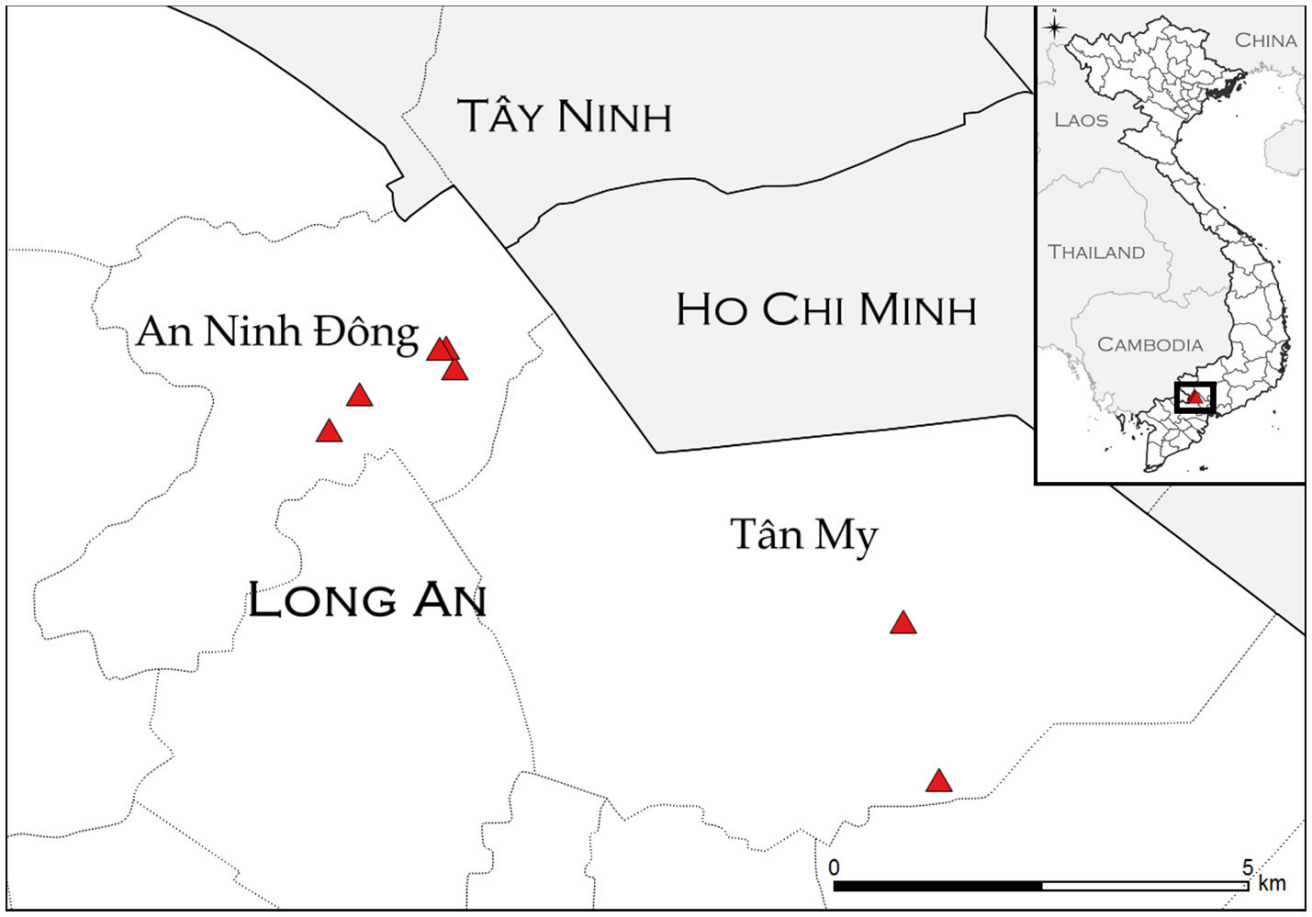
Study sites (triangles) in An Ninh Ðông and Tân Mỹ commune within Long An province. Location information was not available for one farm (AND-14). Inset: location of study sites within Vietnam.

The purpose of the transmission study was to investigate whether FMDV could be transmitted from asymptomatic carrier cattle to naïve sentinel cattle under typical Vietnamese husbandry conditions. The Long An province is located in the Mekong Delta region of southern Vietnam (Figure 1). From September 2012 to March 2013, the average daily temperature was 27.2 degrees Celsius with daily temperatures consistently above 25°. The average relative humidity during the study period was 75%, with relative humidity ranging from 59 to 91% (41). In 2012, ten persistently infected cattle on eight farms in the Long An province were identified by confirmed seroreactivity against FMDV non-structural proteins (NSP) and detection of FMDV RNA in oropharyngeal fluid (OPF) as previously described (33). By administering questionnaires to the animals' owners, it was determined that the most recent outbreaks of clinical FMD had occurred on all farms in 2010 or 2011. For this study, an outbreak was defined as the occurrence of clinical FMD in at least one animal on a farm, as reported by the owner or regional veterinary services. Sixteen FMDV-naïve cattle (demonstrated by the absence of FMDV RNA in OPF and lack of FMDV antibodies in serum) were acquired from local farms and paired with the persistently infected animals to serve as sentinels (Table 1). All naïve cattle were typical outbred *Bos indicus* steers endemic to Vietnam. Naïve cattle were allocated to farms in accordance with logistical limitations of space and resources. On two farms which each had two FMDV carriers, one sentinel animal was paired with each carrier. On the remaining six farms, two sentinel animals were paired with each carrier. Sentinels were housed in direct contact with carrier animals and were obligated to share food and water sources. Additionally, three calves born to FMDV carriers during the study period were kept with their dam and were monitored for seroconversion and detection of FMDV in OPF samples.

**Table 1:** Farm-level animal husbandry practices of premises with confirmed FMDV carrier cattle in Long An province, Vietnam. All farms are in Long An province.

**Farm**	**Total cattle**	**Carrier ID**	**No. Sentinels**	**Breed^†^**	**Housing system**	**Purpose**	**Source of cattle**	**Pasture contact**	**Nearby cattle vaccinated**	**Last FMD vaccine**	**Last FMD outbreak**
AND-05	23	A051 A056	2	H-F *B. indicus*	Pasture, Restraint	Dairy, Beef	Neighboring province	Cattle	Yes	Oct-2011	2010
AND-13	1	A084	3^*^	*B. indicus*	Restraint	Beef	Local	NA	Yes	Oct-2011	2010
AND-14	1	A085	2	*B. indicus*	Restraint	Beef	NA	Cattle, Buffalo	Yes	None	2011
AND-15	4	A086	2	*B. indicus*	Pasture, Restraint	Dairy, Beef	Local	Cattle	Yes	None	2010
AND-19	7	A124	3^*^	H-F	Pasture	Dairy	Local	None	NA	Sep-2011	2010
AND-22	2	A151 A152	3^*^	*B. indicus B. indicus*	Restraint	Beef	NA	Cattle	NA	NA	2010
TM-07	9	B078	2	H-F	Pasture, Restraint	Dairy, Beef	Local	Cattle, Buffalo	No	Jan-2011	2010
TM-31	7	B177	2	*B. indicus*	Restraint	Beef	NA	None	No	Unk	2010

*AND, An Ninh Ðông commune; TM, Tân Mỹ commune*.

* *Denotes a calf was also included in the study as an additional sentinel*.

† *Dairy cattle were Holstein-Friesian (HF) crosses; beef cattle were typical outbred Bos indicus endemic to Vietnam. All sentinel cattle were also B. indicus endemic to Vietnam*.

Serum and OPF samples were collected and screened as previously described (33). Briefly, serum samples were screened for the presence of antibodies against NSPs of FMDV using a 3ABC-ELISA kit (PrioCheck R, Prionics, Netherlands: Product No 7610450) following manufacturer's instructions. OPF samples were collected using a probang cup (9) and screened for the presence of FMDV RNA as described below. Persistently infected animals were identified by serum and OPF samples collected in April 2012. Two additional OPF samples were collected from persistently infected animals in May and July 2012. Sentinel animals were introduced in September 2012, and serum and OPF samples were collected monthly from all animals for 6 months through March 2013.

### Questionnaire on husbandry practices

A questionnaire was distributed to farmers as previously described (33). Briefly, farmers were queried about a series of potential FMD risk factors, including number and type of animals on the farm, type of animal housing (restraint or pasture), animal purpose, source of new animals, history of FMD in the herd (year of last outbreak), vaccination practices, and potential contact with neighboring animals (Table 1). The purpose of this questionnaire in the current study was to collect information which would contribute to understanding of carrier-to-sentinel transmission events, and rule-in or out potential confounding factors such as exposure to other animals in the field.

### Virus isolation

Virus isolation (VI) from OPF samples was performed at the Plum Island Animal Disease Center (PIADC), Greenport, NY, as previously described (12, 42). Briefly, OPF samples were collected and homogenized in minimal essential media containing 25 mM HEPES, and then treated with 1,1,2-trichlorotrifluoroethane (TTE) to dissociate immune complexes. The aqueous phase of the TTE-treated samples was processed through Spin-X filter columns (Costar, Corning, NY, USA), and 250 μl of the filtrate was inoculated into LFBK αVβ6 cell culture (43, 44) for VI as previously described (45). FMDV-positive VI supernatants were confirmed to contain FMDV RNA using quantitative reverse-transcriptase polymerase chain reaction (qRT-PCR).

### FMDV RNA detection and sequencing

All OPF samples were screened using qRT-PCR as previously described (33). Briefly, a 50 μl aliquot was treated for 1 h at 37°C with an enzyme mix (33), after which RNA was extracted and analyzed by qRT-PCR (45). Samples were considered positive when *C*_t_ values were < 40 (45).

The viral RNA from positive samples was further analyzed by RT-PCR and Sanger sequencing as previously described (33). Complete VP1 sequences were obtained using internal sequencing primers specific to the Vietnamese isolates (33). Complete open reading frame (ORF) sequences were obtained from overlapping RT-PCR fragments and sequenced as previously described (46).

### VP1 phylogenetic analysis

FMDV VP1 sequences were obtained from probang samples from 5 of the 10 persistently infected animals in this study. Evolutionary modeling and visualization of the phylogenetic relationship between the viruses was performed using the BEAST v 1.8.4 software suite (47). For regional and temporal context, 39 additional serotype O/ME-SA/PanAsia sequences from GenBank, as well as 3 newly generated sequences [GenBank: MF143572-8] were included in the analysis. For carrier animals with serial isolates (animals B177, A152, and A086), only the earliest dated VP1 sequences were used for estimation of overall substitution rate.

The VP1 tree was created using those clades most commonly sampled amongst all trees after 10% burn-in, as inferred by Bayesian Markov chain Monte Carlo (MCMC) analysis, implemented in BEAST. The nucleotide substitution model used was Tamura-Nei'93 with 4-bin gamma values (the model with the lowest BIC (Bayesian information criterion) as determined using MEGA v 7.0), with unlinked rates between codon positions (48, 49). The molecular clock model was set as uncorrelated (exponential dist.; initial annual rate of 0.01), the tree model set as Bayesian skyline, and tip dates set as approximate or precise (when known) (50, 51). Every 2500th iteration of a 2.5 × 10^7^-step MCMC was sampled.

### Within-host evolution of FMDV

Full ORF sequences were derived from seven sequential OPF samples collected from animal B177 on: June 7, 2012; July 7, 2012; September 4, 2012; October 5, 2012; January 3, 2013; February 4, 2013; and June 17, 2013. To understand the within-host evolution of FMDV in this animal, the seven sequences were analyzed using statistical parsimony, implemented in TCS software (52). The output of the TCS analysis was depicted using PopART (http://popart.otago.ac.nz). Full genome sequences used for this analysis are available [GenBank: MF143579-81].

### Tissue distribution of FMDV

Two carrier animals that had FMDV RNA in OPF samples for the duration of the transmission study were euthanized in June 2013, after the conclusion of this study, for routine human consumption, and tissues were harvested through a limited necropsy procedure at the slaughter facility in order to investigate the anatomic sites of persistent infection. Serum and OPF samples were collected immediately prior to euthanasia. Necropsies were performed immediately after euthanasia and tissue samples were collected from the dorsal soft palate, dorsal nasopharynx (rostral and caudal sub-compartments of both specimens), ventral epiglottis, larynx, palatine tonsil, retropharyngeal lymph node, submandibular lymph node, and popliteal lymph node (Table 2) as previously described (45, 53). Briefly, two 30 mg samples from each tissue were aliquoted into separate screw-cap 1.5 ml tubes and stored at −70°C until processing. Necropsies were performed in Vietnam and samples were shipped to PIADC for analysis.

**Table 2:** Carrier state duration (months) based upon three assumed timepoints in the reported year of most recent FMD outbreak.

Assumed date of infection^*^	**Median**	**Restricted mean (SE)**	**Upper limit of restriction**
January 1 (Beginning)	34.1	33.6 (1.7)	38.4
July 1 (Midpoint)	28.1	27.7 (1.7)	32.4
December 31 (End)	22.2	21.7 (1.7)	26.5

*The first sampling date for carriers was April 13, 2012, and the last sampling date was March 15, 2013*.

* *For each animal, the year of most recent outbreak was known (2010 or 2011), however the month of the outbreak was undetermined*.

Tissue samples were macerated using a TissueLyser bead beater (Qiagen, Valencia, CA), after which 50 μl of each macerate was subjected to RNA extraction followed by qRT-PCR, and the remaining macerate was subjected to virus isolation as described above and previously (45, 53).

### Statistical analysis

Carrier animals were assumed to have been infected during the most recent outbreak of clinical FMD in the herd, and the duration of persistent infection was calculated as the time from the most recent outbreak to the time when the animal stopped shedding viral RNA in OPF (defined as the midpoint between the last positive sample and the third consecutive negative sample). Only the year of the most recent FMD outbreak affecting each herd was provided, and the analysis of carrier state duration was performed using a lagged start date to account for the inherent uncertainty in the date of the most recent outbreak. The duration of persistent infection was calculated using three distinct assumed dates of the most recent FMD infection: January 1st (beginning), July 1st (midpoint), or December 31st (end) of the reported year (Table 2). The values reported are based upon the most recent FMD infection occurring at the midpoint of the year unless otherwise stated. Animals that were still shedding virus at the end of the study period were treated as right censored in the analysis (54). The Kaplan-Meier test was used to estimate the median and restricted mean duration of persistent infection. The log-rank test was used to investigate relationships between the duration of persistent infection and farm size (small or intermediate), primary animal purpose (beef or dairy), and pasture contact with other animals (yes or no). Sentinel animal time-at-risk was calculated as the time from the introduction of the sentinel until extinction of the carrier state of the paired donor animal or the end of the study if the donor remained persistently infected throughout the study. Calf time-at-risk was calculated as the time from when the calf was 3 months old until extinction of the carrier state of the dam or the end of the study. The rationale for this definition was based upon the assumption that maternal antibody protection against FMDV wanes by 3–4 months in calves (55, 56). The incidence rate of infection (number of new infections per sentinel-month at risk) was determined for sentinel animals, including calves. The force of infection [per capita rate at which sentinels become infected (54)] was estimated by maximum likelihood. Assuming a constant force of infection for the duration of the study, the probability of a naïve sentinel animal becoming infected is given by:

(1) $$\begin{array}{r} Prob\left(transmission\right)=1-e^{-\lambda T}, \end{array}$$

where λ is the force of infection and *T* is the duration of contact between the carrier and the sentinel (57). Data analysis was carried out using R version 3.3.2 (R Core Team, 2016) with the survival, epitools, and bbmle packages.

## Results

### Husbandry practices

Ten persistently infected cattle on eight farms were monitored for seroreactivity and detection of FMDV RNA in OPF for 12 months, April 2012–March 2013. The eight farms included in the study were all in the Long An province (Figure 1). Two were in Tân Mỹ commune, and six were in An Ninh Ðông commune. Two sentinel animals were provided to each farm for this study, and three farms each had one calf that was included in the study (Table 1). The median herd size was 5.5 (range 1–23), with four small herds (1–5 animals), three intermediate herds (6–12 animals), and one large herd (>13 animals). The large herd was included with the intermediate herds for analyses. Half were beef herds, and half were dairy or dairy and beef, with housing systems corresponding to animal purpose (Table 1). During the data collection survey conducted in 2012, the year of the most recent FMD outbreak was recorded. The most recent outbreak in one herd was reported to have occurred in 2011, whereas the most recent outbreaks were reported to have occurred in 2010 in the other seven herds. This data is consistent with the records of the Epidemiology Division, Department of Animal Health (DAH), Vietnam.

### Duration of persistent infection and transmission to sentinel cattle

**Figure 2:**
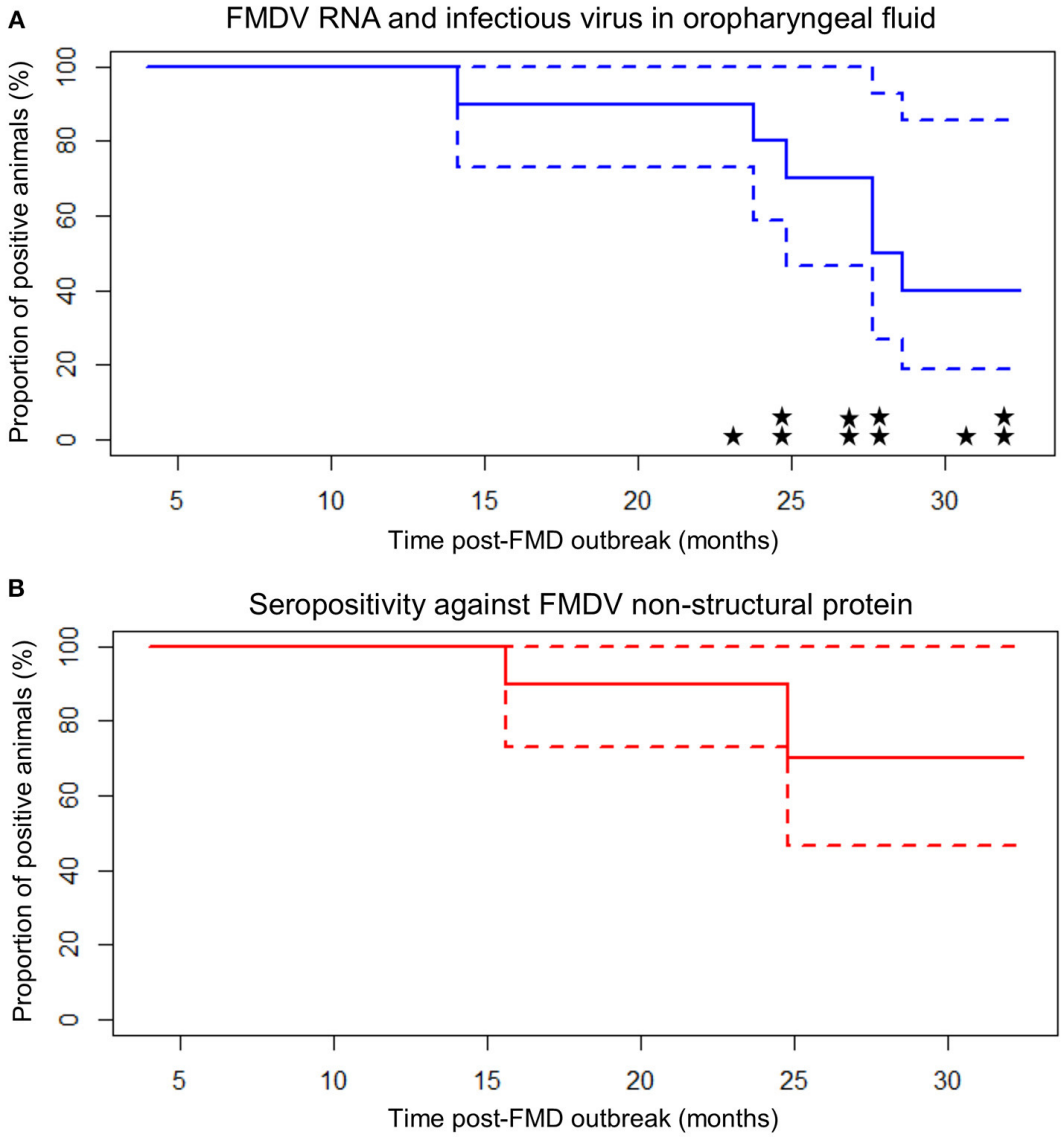
Carrier state extinction curves for 10 cattle persistently infected with FMDV in Long An, Vietnam. Elapsed time (*x*-axis) is from the midpoint of the reported year of FMD outbreak in each animal's resident herd (9 or 21 months prior to the start of sample collection). Dashed lines represent 95% confidence intervals. **(A)** Probang (OPF) samples. Extinction curve is based upon detection of FMDV RNA in oropharyngeal fluid. Stars represent detection of infectious virus (one star = one positive sample). **(B)** Serum samples screened by anti-FMDV non-structural protein competitive ELISA.

Of the ten persistently infected cattle included in the study, nine became infected with FMD in 2010 and one became infected in 2011. Based upon the assumption that animals were infected at the midpoint of the reported outbreak year, the study ended 32.4 months after infection for animals infected in 2010 (Table 2; Figure 2). During the study, the mean duration of anti-FMDV non-structural protein (NSP) antibodies in the serum was 29.2 ± 1.7 (SE) months, and seven cattle remained NSP-seropositive at the end of the study, precluding calculation of the median duration. The median duration of FMDV RNA detection in OPF was 28.1 months, the mean duration was 27.7 ± 1.7 (SE) months, and four cattle remained OPF-positive at the end of the study (Figure 2). Because extinction of the carrier state was not detected in all animals, it is likely that the calculated mean duration of seropositivity and carrier state are underestimated. The rate of decrease in the proportion of persistently infected animals was 0.03 ± 0.005 (SE) per month. The duration of persistent infection was not different between farm size (log-rank *p* = 0.32), animal purpose (log-rank *p* = 0.32), or pasture contact with other animals (log-rank *p* = 0.84), although small sample size limited the ability to detect significant differences.

Sixteen intentionally introduced sentinel cattle and three calves born to persistently infected dams were included in the study as contact exposure sentinels. Based upon the assumption that carrier animals were infected at the midpoint of the reported outbreak year, sentinel cattle were introduced 26.1 months after infection for carriers infected in 2010. One calf was seropositive upon entry into the study at 3 months of age, but was seronegative at all subsequent sampling times, and there was no detection of FMDV in any OPF samples from this calf. The sixteen sentinel cattle and remaining two calves all remained seronegative and there was no detection of FMDV in OPF from any of these animals through the duration of the study. The sentinels and calves contributed a cumulative (combined) total of 54.6 animal-months at risk. The incidence rate for FMDV infection in sentinels and calves was 0 per animal-month at risk, with an upper 95% confidence limit of 0.068 per animal-month at risk. FMDV RNA was isolated from OPF samples from persistently infected animals for a cumulative total of 29.9 months (summed across all carriers) while the carriers were in contact with sentinel animals during the study. The maximum likelihood estimate for the force of infection was 0 per month, with an upper 95% confidence limit of 0.064 per month (0.0021/day).

### VP1 phylogenetic analysis

**Figure 3:**
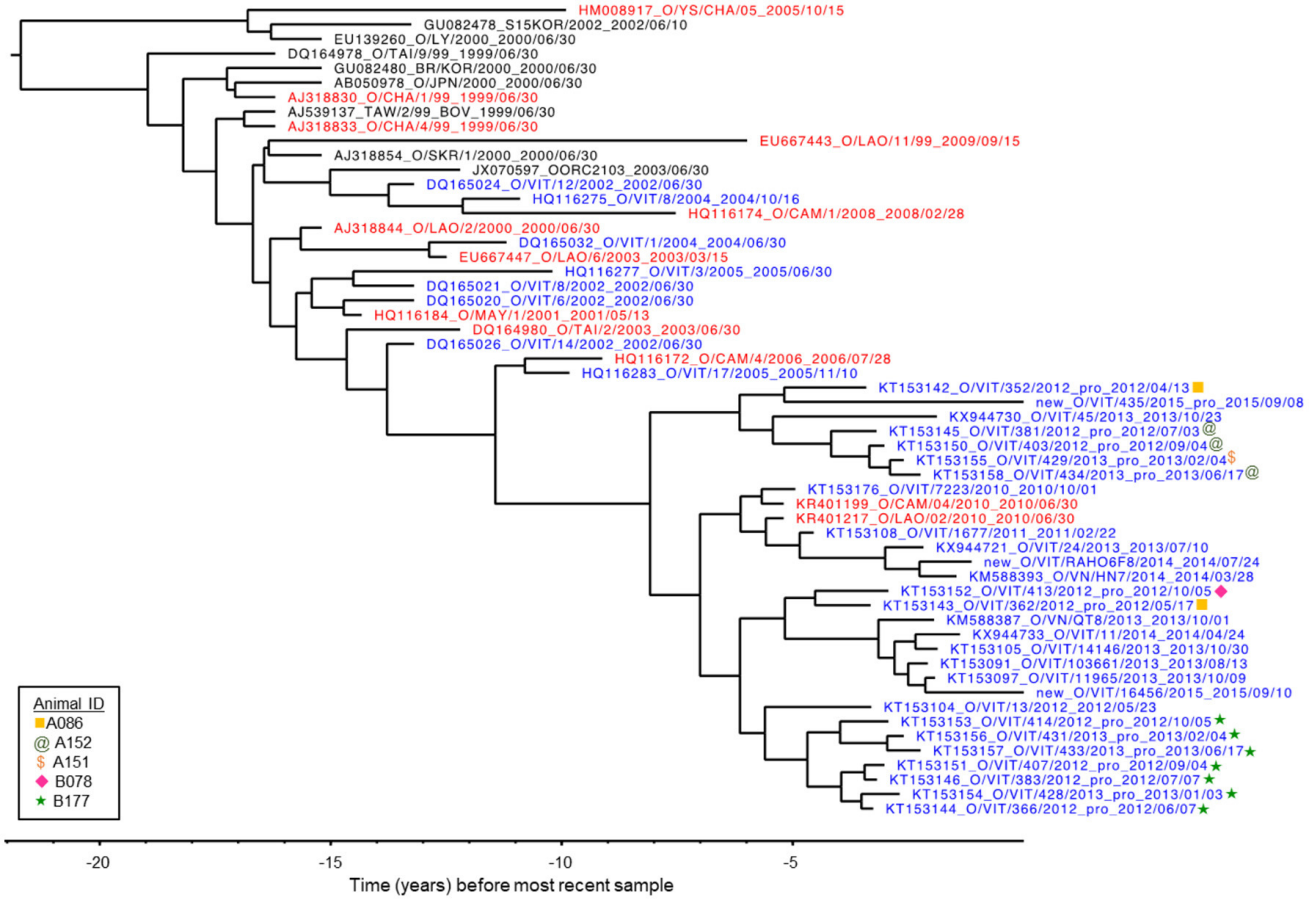
VP1 phylogenetic tree as inferred by Bayesian analysis (BEAST). The tree represents the relationship between the FMDV viruses isolated from five animals in the current study in the context of previously published O/ME-SA/PanAsia VP1 sequences. Three new sequences are also included, labeled “new.” Blue: isolates from Vietnam. Red: isolates from countries neighboring Vietnam (Laos, Cambodia and China). *X*-axis indicates years before most recent sample.

VP1 sequences were obtained from infectious FMDV isolated from 12 OPF samples from four different individuals collected throughout the study, as well as from FMDV RNA obtained directly from two OPF samples from one additional individual. All sequences belonged to the O/ME-SA/PanAsia lineage. GenBank BLAST search as well as Bayesian phylogenetic analysis (Figure 3) indicated that the viruses sampled in the current study were genetically most similar to contemporary FMDV isolates originating from Vietnam, Cambodia, and Laos (33, 34). Furthermore, with the exception of one sample (KT153143), viruses were reliably predicted to segregate into clades according to commune of origin, and viruses obtained from serial samples collected from the same individual formed a monophyletic group (Figure 3; Table 1). BEAST analysis estimated the average rate of VP1 nucleotide substitution to be 5.8 × 10^−3^ substitutions/site/year.

### Within-host evolution of FMDV

**Figure 4:**
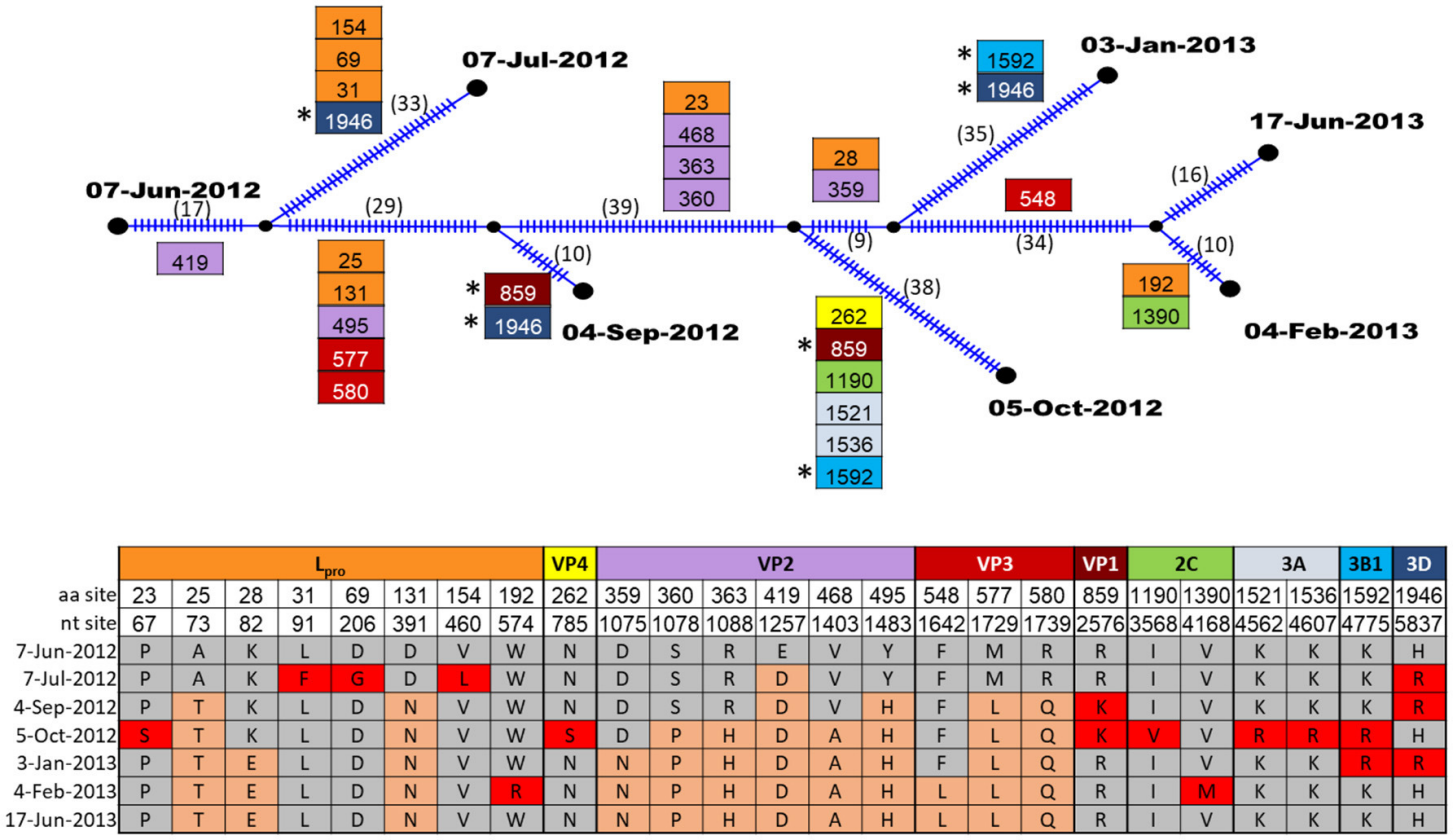
TCS parsimony reconstruction of FMDV ORF sequences from sequential samples of one carrier animal, B177. Ticks in branches and number in parentheses indicate the total number of nucleotide changes inferred by TCS. Boxes indicate non-synonymous changes and their specific aa sites within the protein coding region indicated by color. Boxes are located at the specific branches for changes common to all descendants. (*) indicate identical non-synonymous changes that occurred in more than one descendant in intermediate samples. The table shows the amino acid changes at specific nucleotide and amino acid sites within protein coding regions. Amino acids that were present in the first sample are indicated by gray shading, amino acid mutations which became fixed in subsequent samples are shaded orange, and amino acid mutations that were present transiently, but reverted are indicated in red shading.

The evolution of FMDV within one persistently infected cow (B177) over the course of one year was investigated through analyses of full open reading frame (ORF) sequences of 7 serial OPF samples. For this animal, the samples from which virus sequences were obtained were estimated to span 23–35 months post infection. Evolutionary reconstruction of the serial virus sequences by the algorithm implemented in Templeton-Crandall-Sing analysis (TCS) (52) indicated that several nucleotide changes had occurred throughout the genome (Figure 4). In total, 25 sites had non-synonymous mutations, specifically in L_pro_ (8 sites), VP4(1), VP2(6), VP3(3), VP1(1), 2C(2), 3A(2), 3B(1), and 3D(1). Most of these changes occurred at one point throughout the study period, and were fixed (evidenced by the presence of the changes in all following samples), however all segments except VP2 and VP3 had changes that were present transiently, but reverted to the ancestral state in subsequent samples (Figure 4). Pairwise comparison between the first virus collected (07-Jun-2012; estimated 23 months post-outbreak) and each of the subsequent samples indicated increasing quantities of nucleotide and amino acid changes as the time between samples increased (Table 3). Most of the nucleotide changes (*n* = 133) observed between the first and the last samples occurred in L_pro_ (*n* = 19), VP2 (*n* = 18), VP3 (*n* = 12), VP1 (*n* = 16), 2C (*n* = 18), 3C (*n* = 12) and 3D (*n* = 26), whereas non-synonymous changes (all samples combined compared to the first sample) were found mostly in L_pro_ (*n* = 18), VP4 (*n* = 1), VP2 (*n* = 26), VP3 (*n* = 12), and less frequently in VP1 (*n* = 2), 2C (*n* = 2), 3A (*n* = 2), 3B (*n* = 2), and 3D (*n* = 3).

**Table 3:** Number of nucleotide (nt) and amino acid (aa) substitutions in FMDV ORF protein coding segments obtained from sequential samples of persistently infected cow B177.

		**Segment (nucleotide length)**												
**Date**		**L_pro_**	**VP4**	**VP2**	**VP3**	**VP1**	**2A**	**2B**	**2C**	**3A**	**3B**	**3C**	**3D**	**FLS**
		**(603)**	**(255)**	**(654)**	**(660)**	**(633)**	**(54)**	**(462)**	**(954)**	**(459)**	**(213)**	**(639)**	**(1410)**	**(6996)**
7-Jul-12	nt	6	6	9	3	1	0	0	4	5	0	5	11	50
	aa	3	0	1	0	0	0	0	0	0	0	0	1	5
4-Sep-12	nt	12	1	9	6	6	0	3	3	1	1	6	8	56
	aa	2	0	2	2	1	0	0	0	0	0	0	1	8
5-Oct-12	nt	16	5	16	8	13	0	5	14	5	3	11	20	116
	aa	3	1	5	2	1	0	0	1	2	1	0	0	16
3-Jan-13	nt	19	2	19	15	8	0	6	13	4	2	16	25	129
	aa	3	0	6	2	0	0	0	0	0	1	0	1	13
4-Feb-13	nt	19	2	17	11	16	0	6	19	3	1	13	26	133
	aa	4	0	6	3	0	0	0	1	0	0	0	0	14
17-Jun-13	nt	19	4	18	12	16	0	4	18	3	1	12	26	133
	aa	3	0	6	3	0	0	0	0	0	0	0	0	12

*Numbers of substitutions are relative to the first virus isolated from this animal on Jun-06-2012*.

### Tissue distribution of FMDV

The tissue-specific distribution of FMDV and viral RNA was investigated in two carriers from which FMDV RNA and infectious virus could still be recovered in OPF samples at the conclusion of the study (Table 4). Of 10 tissues that were screened from each animal, FMDV RNA was detected by qRT-PCR in the laryngeal mucosa in both animals, as well as in the caudal dorsal soft palate in one animal (Table 4). However, infectious virus was not retrieved from any tissue specimen sampled.

**Table 4:** Results of FMDV qRT-PCR and virus isolation on tissues from two persistently infected cattle.

	**Animal**	
**Tissue**	**B177**	**A152**
**NASOPHARYNX/LARYNX**		
Dorsal soft palate (rostral)	Neg	Neg
Dorsal soft palate (caudal)	POS	Neg
Dorsal nasopharynx (rostral)	Neg	Neg
Dorsal nasopharynx (caudal)	Neg	Neg
Epiglottis (ventral)	Neg	Neg
Larynx	POS	POS
**TONSILS AND LYMPH NODES**		
Palatine tonsil	Neg	Neg
Retropharyngeal LN	Neg	Neg
Submandibular LN	Neg	Neg
Right popliteal LN	Neg	Neg
OPF	*POS*	*POS*
NSP ELISA	POS	POS

*OPF and serum samples were collected on the day of necropsy. Italics indicate samples positive for virus isolation*.

## Discussion

The existence of persistently infected FMDV carriers and the potential risk for transmission these animals represent have led authorities to develop policies aimed at restricting international trade of animals and animal products from FMD endemic regions and regions where FMDV vaccination is practiced, as well as to recommend euthanasia of all infected animals when outbreaks occur in FMD-free regions (58). The perceived risk posed by FMDV carriers is one of the reasons that FMD-free countries typically control outbreaks using depopulation of affected and vaccinated herds as a first-line response. In FMD-endemic regions, FMD-outbreak premises are often quarantined, but the appropriate duration of quarantine has not been empirically established since the risk posed by carrier ruminants is not well-defined. Depopulation, quarantine, and trade restrictions have a high economic impact on FMD-affected areas without a guarantee of preventing spread to FMD-free areas (4, 7, 59). Thus, an improved understanding regarding the risk of transmission from carriers and the duration of the carrier state is needed to inform FMD control and animal health strategies worldwide.

In the current study, seroreactivity and detection of FMDV RNA in OPF in 10 persistently infected cattle on eight farms were monitored over 12 months, April 2012–March 2013. It was assumed that persistently infected FMDV carriers had been infected during the most recent FMD outbreak in the herd. However, only the year of the most recent outbreak was available, and therefore our results must be interpreted as approximations. We have determined an average duration of persistent infection of 27.7 months, with estimates ranging between 21.7 and 33.6 months based on assumptions about the date of infection. In contrast, previous studies have reported shorter maximum durations, between 10 and 24 months in cattle (10, 16, 17, 19), and many cattle cleared the infection within 6 months (8, 16, 18, 20). In the current study, carrier cattle that cleared the infection within 6 months would have been excluded due to the timing of initiation of sampling, which would have biased the estimate of the average duration of persistent infection, but not the maximum duration, toward a longer duration.

This study suggests that a subset of FMDV carrier cattle may maintain persistent infection longer than has previously been reported. Based upon the owner-reported dates of the last FMDV outbreaks amongst the cattle in this study, 9 of 10 cattle had already been persistently infected for at least 12 months at the start of the study. We have documented that 6 of 10 cattle maintained persistent infection for 14–28 months post-infection, and 4 of 10 animals had not terminated infection at 32 months. Previous reports indicate only a small fraction of persistently infected cattle maintain the infection longer than 12 months. For example, Hedger (21) found a 38% prevalence of carriers 6 months after a serotype SAT1 FMDV outbreak, but only 5.4% prevalence 12 months after the outbreak. Similarly, a modeling-based approach to FMDV persistence, using cross-sectional data from an area where serotypes A, O, and SAT2 are endemic, estimated a 0.7% probability of recovering virus from cattle more than 12 months after an outbreak (60). However, these reports do not document the duration of persistent infection in the fraction of animals that maintain persistent infection beyond 12 months. In contrast, Hayer et al. (17) reported an average carrier state duration of 13.1 months with complete herd-level termination at 15–19 months in cattle naturally infected with serotype O/ME-SA/Ind-2001d strain of FMDV.

The longer duration of persistent infection in this study compared to previous reports may reflect differences in virus (serotype, strain or virulence), host (genetic differences, nutritional or immunological status), environment (climate, mineral intake), and differences between field and experimental conditions. Environmental conditions are important for FMDV survival outside the host and may affect transmission under natural conditions. In one study, low-lying areas in Vietnam had a higher economic impact from FMD, compared to midland and highland regions (61), suggesting differences in ecology may favor FMDV survival, resulting in a higher incidence of FMD in lowland areas.

In contrast to field studies, environmental conditions are controlled in many experimental studies, reducing variation in FMDV survival and transmission due to environmental differences. Many reports of cattle clearing the infection within 6 months were experimental studies (8, 18, 20), which utilize challenge viruses and transmission routes that are distinct from natural infections under field conditions. However, the longer duration reported in the current study may also be due in part to the uncertainty in the reported date of the most recent FMD outbreak in the herd. To account for this uncertainty, we performed the analysis with lagged start dates to provide the minimum and maximum possible duration given the year of the outbreak.

An important potential confounding factor in this study is the possibility that the carrier cattle had become subclinically infected with a new FMDV during the study. Subclinical infections may be important in FMDV epidemiology in endemic regions (62), and subclinical infections in our study would have gone unnoticed by the owners. This would have had the effect of biasing toward longer apparent duration of the carrier state. However, serology and OPF analyses confirmed there was no FMDV infection in any of the naïve sentinel cattle, indicating no incursion of a novel FMDV during the study. Additionally, if novel FMDVs had been introduced into the herd during the study period, clinical cases would have been expected amongst some of the vaccinated carriers and most or all of the naïve sentinel animals. No such clinical cases occurred.

To our knowledge, this is the first report of an experimental investigation of transmission from carrier to naïve cattle under typical husbandry conditions. During the study, FMDV was not detected in any OPF samples from sentinel animals or calves, and none of the sentinels seroconverted. Transmission to sentinels did not occur despite repeated isolation of FMDV and viral RNA from OPF samples from persistently infected carriers. Our finding of lack of transmission is consistent with previous studies investigating transmission from carrier cattle to sentinel cattle [reviewed in Tenzin et al. (37)]. In the current study, the upper 95% confidence limit for the force of infection was 0.064 per month, which is consistent with two previous studies (22, 37), and indicates that at most, one transmission event would be expected to occur in 15.6 months of contact time. This study supports previous findings of a low (or absent) risk of transmission from carrier to naïve cattle. One calf was seropositive upon entry into the study (at 3 months of age), but was seronegative on subsequent samples for the duration of the study, and FMDV RNA was never recovered from OPF samples from this individual. The initial positive result for this calf was likely due to the presence of maternal antibodies. The limited data reported herein suggests that transmission from carrier cattle to calves does not appear to be important in the epidemiology of FMDV in cattle. This is in contrast to African buffalo, in which transmission from carrier animals to calves after maternal antibodies have waned is reported to be an important mechanism of virus maintenance in this species (14, 28, 63).

Sentinel cattle were introduced approximately 26 months after infection of the carriers, based on estimating carrier infection at the midpoint of the reported year; therefore this study reflects the potential for transmission after prolonged persistent infection. The current study suggests a very low risk of transmission to naïve animals introduced onto farms more than 2 years after an outbreak, despite the presence of persistently infected cattle from which FMDV could still be recovered. However, our study design could not address the potential of transmission during the early portion of the carrier phase. Previous studies have suggested that transmission potential is low during that period as well (22, 37).

Phylogenetic analysis of VP1 coding sequences indicated the viruses obtained in this study were most closely related to contemporary viruses collected in Vietnam, suggesting the O/ME-SA/PanAsia lineage was circulating endemically in the region during the study period. For individuals from which serial isolates were obtained, phylogenetic analysis also supported the owner-reported timing of infection in 2010. For example, the most recent common ancestor of the isolates collected from animal B177 was estimated to occur approximately 5 years prior to the most recent isolates in the analysis (collected in 2015). The phylogenetic inferences and genome-wide substitution analysis support the interpretation that the multiple isolates from that animal represent within-host evolution of an earlier infection rather than serial superinfections. This phenomenon is consistent with similar findings of FMDV within-host evolution under natural conditions in Pakistan (64).

We recovered FMDV and acquired full ORF sequences of samples serially collected over a 1-year period from one of the FMDV-persistently infected animals. Serial ORF sequences collected from a persistently infected animal in the field are unprecedented to our knowledge. Analysis of the evolution of FMDV within this animal during the study demonstrated that the proteins that underwent the most non-synonymous changes were the leader proteinase L_pro_ and capsid proteins VP2 and VP3. L_pro_ is a well-characterized determinant of virulence (65–67). Similar to our findings in one animal, a previous study analyzing full ORF sequences of viruses from groups of experimentally infected, vaccinated and unvaccinated carrier animals found amino acid changes within L_pro_, VP3, VP2, VP1, 2C, 3A, 3B, and 3D (22). Additional specific amino acid changes and positive selection within the known FMDV antigenic sites of viruses recovered from carrier animals have also been reported (25, 26, 34), whereas the capsid proteins have a critical role as anti-receptors in host cell binding (68) as well as generating the most relevant host immune response (69, 70). The accrual of these amino acid substitutions may alter these proteins resulting in improved fitness and enabling immune evasion. Further investigation, including analyses of serial sequences from greater quantities of animals, is needed to elucidate whether mutation in these regions is a commonality of adaptation of FMDV across persistently infected hosts and viral strains.

Burrows (20) was the first to show experimentally that the tissues of the nasopharynx were the anatomic sites of FMDV persistence. Subsequent studies have further localized persistent FMDV infection to specialized segments of follicle-associated epithelium of the nasopharynx (12, 42) or associated lymphoid tissue (15, 71). In the current study, FMDV RNA was recovered from the laryngeal mucosa of two persistently infected cattle and the caudal dorsal soft palate of one of these animals, suggesting tissue localization under natural infection is similar to distribution in experimental infections. Although infectious virus was not recovered from any of the tissue samples, FMDV was isolated from OPF samples collected immediately prior to necropsy, indicating the presence of replicating virus in these carrier animals at the time of necropsy. FMDV is not evenly distributed within tissues (42), and the specific sections collected for virus isolation may have contained little or no virus even though adjacent sections may have been infected. Additionally, it is possible that sample preservation failures may have occurred during the transfer of samples from DAH, Vietnam to PIADC, USA, which would have decreased the amount of viable virus in the sample, thereby decreasing the likelihood of successful virus isolation.

Despite decades of research investigating the FMD carrier state, the answers to two persistent questions remain unsatisfactorily resolved: What is the duration of persistent infection? And, what is the risk of transmission from a persistently infected animal? The current study contributes to these knowledge gaps, albeit on a very limited scale, and under the specific conditions of the study, including persistent infection with FMDV serotype O/ME-SA/PanAsia and typical Vietnamese husbandry practices, climate, and cattle breeds. These data suggest that the duration of persistent infection in some animals may be longer than previously documented, but the risk of transmission is exceedingly low. Additionally, this study shows that the sites of viral persistence in tissues of the pharynx are similar to what has been described under experimental conditions. This study also contributes to knowledge of within-host evolution of FMDV. Although care should be taken interpreting the current small study, these results will contribute to informing policy decisions regarding FMDV carriers. Further studies are needed to investigate the duration of persistent infection under typical husbandry practices and the role of carriers in FMDV epidemiology in endemic areas.

## Data availability statement

The datasets analyzed during the current study are available from the authors upon reasonable request.

## Author contributions

MB performed the statistical analysis and drafted the manuscript. LV, BH, NP, VH, and NL planned and executed the sample acquisition in Vietnam and conducted the primary screening of samples. SP oversaw and executed the sequence acquisition studies, and helped to draft the manuscript. BB performed the ORF analysis, contributed to the statistical analysis, and helped to draft the manuscript. EH and GS oversaw and executed sample processing and contributed to sequence acquisition. CS carried out the tissue studies, and contributed to drafting the manuscript. IF performed the VP1 analysis, and helped to draft the manuscript. AD and KV contributed to the statistical analysis, and helped to draft the manuscript. LR participated in the design and coordination of the study. DD participated in the design and coordination of the study, and contributed to sample acquisition and analysis in Vietnam. JA conceived the study, participated in the design and coordination, and helped to draft the manuscript. All authors read and approved of the final manuscript.

### Conflict of interest statement

The authors declare that the research was conducted in the absence of any commercial or financial relationships that could be construed as a potential conflict of interest.
